# Real-World Survival Outcomes of Glioblastoma Patients Under Universal Temozolomide Access in Uruguay: A Retrospective Cohort Study (2009–2018) and Implications for Health Policy

**DOI:** 10.7759/cureus.113315

**Published:** 2026-07-24

**Authors:** Maria Massia, Robinson Rodriguez, Sebastian Ximenez

**Affiliations:** 1 Rehabilitation Medicine, Hospital Rio Carrion, Palencia, ESP; 2 Oncology, Hospital de Clínicas "Dr. Manuel Quintela", Montevideo, URY; 3 Oncology, Hospital Central de las Fuerzas Armadas, Montevideo, URY; 4 Oncology, Hospital de Clinicas "Dr. Manuel Quintela", Montevideo, URY

**Keywords:** approved car-t therapies, glioblastoma, immune checkpoint inhibition, national resource fund, nivolumab plus relatlimab, public health policy, radiotherapy, survival, temozolomide, uruguay

## Abstract

Background

Glioblastoma (GB) is the most aggressive primary brain tumor in adults and is traditionally associated with a poor prognosis. In 2009, Uruguay’s National Resource Fund (Fondo Nacional de Recursos [FNR]) began guaranteeing universal public funding for standard temozolomide (TMZ), establishing an accessible standard of care. This study evaluates the long-term real-world survival impact of that policy and discusses its implications for the future integration of high-cost immunotherapies.

Methods

We conducted a retrospective observational cohort study of adult patients with histologically confirmed supratentorial hemispheric GB who completed initial diagnosis and treatment between January 2009 and December 2017 at the Neuro-Oncology Unit of the Hospital de Clínicas “Dr. Manuel Quintela”, Montevideo, Uruguay. Follow-up extended through December 2018. Overall survival (OS) was estimated using the Kaplan-Meier method, and the study was reported in accordance with the STROBE guidelines.

Results

A total of 32 patients met the inclusion criteria. Mean age was 50.7 ± 15.3 years (range: 19-75 years), and 62.5% (n = 20) were male. Gross total resection was achieved in 56.3% of cases. Median OS for the entire cohort was 17 months (95% CI: 7.0-26.9). Patients who completed the full adjuvant Stupp regimen achieved a significantly longer median OS than those with incomplete treatment (24 vs. 11 months; log-rank p = 0.023).

Conclusions

Universal public funding for TMZ in Uruguay has achieved real-world outcomes consistent with international benchmarks. However, the biological ceiling of the Stupp regimen underscores the urgent need for molecular profiling. As the therapeutic landscape shifts toward ultra-high-cost immunotherapies, establishing this historical baseline is essential for the development of sustainable, equity-driven health policies in middle-income countries.

## Introduction

Glioblastoma (GB) remains the most devastating primary malignancy of the central nervous system. Despite decades of multimodal treatment consisting of surgical resection followed by the standard Stupp protocol of adjuvant temozolomide (TMZ)-based chemoradiotherapy, survival has historically stagnated, with a median overall survival (OS) of 12-15 months in controlled clinical trials [1].

In Latin American healthcare systems, translating these protocols into real-world practice often exposes profound challenges to therapeutic equity. Uruguay established a pioneering regional policy through its National Resource Fund (Fondo Nacional de Recursos [FNR]), which has guaranteed universal public funding for oral TMZ since 2009 [2]. This policy aimed to eliminate financial toxicity and ensure equitable access to the standard of care.

The objective of this study was to perform a comprehensive survival analysis of patients with hemispheric GB treated at the Neuro-Oncology Unit at the Hospital de Clínicas “Dr. Manuel Quintela” in Montevideo, Uruguay, between 2009 and 2017 [3]. By establishing this long-term statistical baseline, we aimed to provide the real-world evidence necessary to evaluate the impact of historical public coverage - a prerequisite for regional health systems planning the future integration of emerging, ultra-high-cost biological therapies.

## Materials and methods

Study design and patient selection

This retrospective, observational cohort study was conducted using clinical records from the Neuro-Oncology Unit at the Hospital de Clínicas “Dr. Manuel Quintela”. The study was designed and reported in accordance with the Strengthening the Reporting of Observational Studies in Epidemiology (STROBE) guidelines. We screened all clinical records of adult patients with histologically confirmed supratentorial hemispheric GB who were initially evaluated between January 2009 and December 2017. Administrative and clinical follow-up for survival status extended through December 31, 2018.

Inclusion criteria were adult patients (≥18 years) with histologically confirmed supratentorial hemispheric GB who received standard multimodal frontline therapy. Frontline protocol compliance was defined as a minimum radiotherapy dose of 40 Gy delivered concurrently with daily TMZ, followed by at least three cycles of adjuvant TMZ maintenance. Patients who received radiotherapy or chemotherapy alone, who received no tumor-directed treatment, or who completed their protocol at another institution were excluded.

Treatment protocol and operational definitions

Extent of surgical resection was categorized as gross total resection (GTR, R0) when postoperative neuroimaging confirmed macroscopically complete tumor removal; subtotal resection (STR, R1-R2) included cases with residual tumor and diagnostic stereotactic biopsies. Standard protocol compliance was defined as concurrent chemoradiotherapy - fractionated radiotherapy (60 Gy over six to seven weeks) plus concurrent daily oral TMZ (75 mg/m²) - followed by adjuvant maintenance, defined as oral TMZ (150-200 mg/m²) for five consecutive days every 28 days for a planned six cycles. OS was defined as the interval from primary surgical intervention to death from any cause, or to the last verified clinical follow-up for censored patients.

Statistical analysis

Continuous variables are expressed as means ± standard deviation (SD), and categorical variables as frequencies and percentages. OS probability was estimated using the Kaplan-Meier method, and differences between groups were assessed with the log-rank test. Univariate and multivariate survival analyses were performed using the Cox proportional hazards model. Given the limited sample size, the multivariate model was restricted to four pre-specified clinical variables to avoid overfitting, following the events-per-variable rule. Statistical significance was set at a two-tailed p < 0.05. All analyses were performed using STATA version 12.0 (StataCorp LLC, College Station, TX).

Ethical considerations

The study protocol was reviewed and approved by the Institutional Ethics Committee of the Hospital de Clínicas “Dr. Manuel Quintela” (approval number CEI-HC-2019-04). Informed consent was waived given the retrospective, anonymized nature of the study. The research was conducted in accordance with the Declaration of Helsinki. All authors confirm that this study involved human participants and their clinical data.

## Results

Patient characteristics and demographics

Fifty clinical records were screened, of which 32 (64%) patients met the inclusion criteria. The remaining 18 patients were excluded because of poor baseline performance status precluding treatment (n = 7), rapid clinical progression before protocol initiation (n = 5), or protocol completion outside our unit (n = 6).

Among the 32 patients analyzed, mean age at diagnosis was 50.7 ± 15.3 years (range: 19-75 years), and 62.5% (n = 20) were male. The most frequent comorbidities were arterial hypertension (37.5%), and tobacco use (25%). Clinical presentation was dominated by motor deficits (50%) and tonic-clonic seizures (50%). Baseline characteristics are detailed in Table 1.

**Table 1:** Baseline demographic and clinical characteristics of the patient cohort (N = 32) *Comorbidities. †Clinical presentation at onset. ‡Tumor topography/distribution. Percentages may sum to more than 100% because patients could present with more than one comorbidity, clinical feature, or topographic site.

Characteristic	Frequency (n)	Percentage (%) / mean ± SD
Sex: male	20	62.5%
Sex: female	12	37.5%
Age at diagnosis, years	-	50.7 ± 15.3
Arterial hypertension*	12	37.5%
Tobacco use (smoking)*	8	25%
Heart failure*	3	9.4%
Myocardial infarction*	2	6.3%
Arrhythmia/dyslipidemia/allergies*	3	9.3%
No comorbidities recorded	8	25%
Motor deficit†	16	50%
Tonic-clonic seizures†	16	50%
Headache†	9	28.1%
Intracranial hypertension syndrome†	3	9.4%
Other focal neurological deficits†	6	18.8%
Parietal lobe‡	13	40.7%
Frontal lobe‡	10	31.2%
Temporal lobe‡	9	28.1%
Occipital lobe‡	3	9.4%
Basal ganglia‡	3	9.4%
Left hemisphere	17	53.1%
Right hemisphere	14	43.8%
Bilateral	1	3.1%

Therapeutic interventions and survival metrics

Maximal safe neurosurgical cytoreduction achieved GTR in 56.3% (n = 18) of patients. The full planned radiotherapy dose (60 Gy) was completed by 90.6% (n = 29) of the cohort, and 68.8% (n = 22) completed all six planned cycles of adjuvant TMZ.

Over a maximum follow-up of 107 months, 26 deaths were recorded. Median OS for the entire cohort was 17 months (95% CI: 7.0-26.9). Patients who completed the full Stupp regimen achieved a significantly longer median OS than those with incomplete maintenance therapy (24 vs. 11 months; log-rank p = 0.023). Long-term survival analysis showed that 15.6% (n = 5) of patients survived to three years and 3.1% (n = 1) survived to five years. Clinical characteristics of the five long-term survivors are summarized in Table 2.

**Table 2:** Clinical characteristics of long-term survivors exceeding the three-year threshold (n = 5) GTR, gross total resection; STR, subtotal resection

Clinical factor	Patient 3	Patient 13	Patient 18	Patient 19	Patient 24
Sex	Female	Female	Male	Male	Male
Age at diagnosis, years	47	27	34	37	19
Relevant comorbidities	Hypertension	Tobacco use	Other	Tobacco use	None
Anatomical location	Parietal	Frontal	Parietal	Frontal	Basal ganglia
Tumor lateralization	Left	Bilateral	Left	Right	Right
Extent of surgery	GTR (R0)	STR (R1)	GTR (R0)	STR (R1)	STR (R1)
MGMT/IDH status	Unknown	Unknown	Unknown	Unknown	Unknown
Stupp regimen compliance	Complete	Complete	Complete	Complete	Complete
5-year survival confirmed	No	No	Yes	No	No

Prognostic determinants (Cox regression)

In the multivariate model, protocol compliance was the only independent predictor of OS: patients who completed the full regimen had a 65% reduction in the risk of death (HR = 0.35; 95% CI: 0.14-0.86; p = 0.021). Extent of resection showed a trend toward improved survival but did not reach statistical significance (Table 3).

**Table 3:** Cox proportional hazards regression models for overall survival (N = 32) Statistically significant values (p < 0.05) are presented in bold. HR, hazard ratio; CI, confidence interval; GTR, gross total resection; STR, subtotal resection

Variable	Univariate HR (95% CI)	p-Value	Multivariate HR (95% CI)	p-Value
Age at diagnosis (per year)	1.02 (0.98–1.07)	0.240	1.01 (0.96–1.06)	0.530
Age at diagnosis (≥70 vs. <70 years)	0.68 (0.39–1.15)	0.110	0.74 (0.41–1.32)	0.280
Extent of resection (GTR vs. STR)	0.76 (0.32–1.81)	0.597	0.81 (0.34–1.95)	0.640
Protocol adherence (complete vs. incomplete)	0.38 (0.16–0.89)	0.025	0.35 (0.14–0.86)	0.021

Safety and toxicity profile

During the concurrent phase, grade 1-2 non-hematological toxicities occurred in 69.5% of evaluable patients, most often asthenia and nausea. Severe (grade 3) hematological toxicities were limited to isolated cases of anemia and thrombocytopenia (3.12% each); adverse events during the concurrent phase are presented in Table 4. During the adjuvant phase, non-hematological toxicities were recorded in 53.1% of patients; hematological toxicity led to dose reduction in one (3.12%) patient and permanent discontinuation in two (6.24%) patients.

**Table 4:** Non-hematological and hematological toxicity during the concurrent chemoradiotherapy phase (N = 32) Non-hematological and hematological toxicity data were unavailable for 9 of the 32 patients; percentages are calculated for the full cohort (N = 32).

Adverse event	n	%
Grade 2 asthenia	10	31.25%
Grade 2 nausea	6	18.75%
Grade 1 nausea	4	12.50%
Grade 1 asthenia	3	9.38%
Grade 2 vomiting	2	6.25%
Grade 1 vomiting / diarrhea / dermatologic rash	3	9.38%
Grade 2 diarrhea	1	3.12%
Urinary tract infection / headache (ungraded)	2	6.25%
Not recorded (non-hematological)	9	28.13%
Grade 2 neutropenia	8	25%
Grade 2 thrombocytopenia	6	18.75%
Grade 1 thrombocytopenia	3	9.38%
Grade 1 neutropenia / grade 1 anemia	4	12.50%
Grade 3 thrombocytopenia (severe)	1	3.12%
Grade 3 anemia (severe)	1	3.12%
Grade 2 lymphopenia	1	3.12%
Grade 2 anemia	1	3.12%
Not recorded (hematological)	9	28.13%

In the adjuvant maintenance phase, non-hematological toxicities were recorded in 53.1% of patients, most often grade 2 asthenia (28.1%) and grade 2 nausea (21.8%). Grade 4 thrombocytopenia occurred in a single patient (3.12%), and grade 3 lymphopenia was recorded in another patient (3.12%). Hematological toxicity led to a dose reduction in one (3.12%) patient and permanent discontinuation in two (6.24%) patients during the standard 150-200 mg/m² maintenance cycles; adjuvant-phase toxicities are summarized in Table 5.

**Table 5:** Non-hematological and hematological toxicity during the adjuvant maintenance phase (N = 32) Non-hematological toxicity data were unavailable for 5 of the 32 patients; hematological toxicity data were available for all 32 patients. Percentages are calculated for the full cohort (N = 32).

Adverse event	n	%
Grade 2 asthenia	9	28.10%
Grade 2 nausea	7	21.84%
Grade 2 vomiting	5	15.60%
Grade 2 diarrhea	4	12.50%
Grade 1 nausea	2	6.24%
Grade 1 asthenia / grade 1 diarrhea / seizures	3	9.36%
Not recorded (non-hematological)	5	15.60%
Grade 2 thrombocytopenia	5	15.60%
Grade 1 thrombocytopenia	3	9.36%
Grade 2 neutropenia	3	9.36%
Grade 1 neutropenia / grade 2 anemia	4	12.48%
Grade 1 anemia	1	3.12%
Grade 3 lymphopenia	1	3.12%
Grade 4 thrombocytopenia (life-threatening)	1	3.12%

Second- and third-line treatment

Following confirmed tumor progression or local recurrence, second-line treatment was offered to 43.8% (n = 14) of the cohort. Twelve (37.5%) patients underwent a second surgical intervention (all subtotal re-resections), and nine received additional tumor-directed treatment. Five (15.6%) patients received second-line salvage chemotherapy with biodegradable carmustine wafers for an average of two cycles. Other second-line modalities included fractionated re-irradiation (n = 1), irinotecan plus bevacizumab (n = 1), TMZ plus bevacizumab (n = 1), carboplatin monotherapy (n = 1), and carboplatin plus etoposide (n = 2).

A third line of treatment, consisting of a third subtotal neurosurgical cytoreduction, was accessible for 9.4% (n = 3) of patients; postoperative third-line systemic chemotherapy consisted of carmustine (n = 1) or carboplatin monotherapy (n = 1). At the study’s administrative census date, 21.9% (n = 7) of patients remained alive.

## Discussion

This retrospective study describes the real-world survival determinants of patients with hemispheric GB treated at a specialized center in Uruguay. The cohort’s median OS of 17 months aligns with the classical international benchmark established by Stupp et al. (14.6 months) [1] and with historical regional data (18 months) reported by a local cohort [3]. This survival estimate represents a substantial improvement over the historical baseline at our institution during the pre-TMZ era (1980-2000), when median OS was approximately 11 months [3], and highlights the impact of equitable public health policy, specifically universal public coverage of oral TMZ under the FNR since 2009 [2].

Nonetheless, translating these controlled-trial protocols into real-world practice reveals a persistent biological ceiling. Although complete adherence to the frontline Stupp regimen significantly extended median survival to 24 months, long-term outcomes remained limited, with only 3.1% of patients reaching five-year survival.

Study limitations and molecular profiling constraints

A primary limitation of this retrospective analysis is the absence of advanced molecular characterization, including MGMT promoter methylation status and IDH1/2 mutation status [4]. During the study period (2009-2017), these biomarkers were not systematically integrated into local public health diagnostic pathways because of structural resource limitations; MGMT status was known for only one patient in our sample. The absence of these markers precludes advanced prognostic stratification and limits our ability to evaluate inherent resistance to alkylating agents. In addition, the small sample size (N = 32) limits the statistical power of the multivariate model, and the hazard ratios should be interpreted with caution. Future regional studies should systematically incorporate molecular profiling in accordance with current World Health Organization classifications [4].

Health policy implications for the immunotherapy era

The international GB treatment landscape is shifting from conventional cytotoxic regimens toward advanced, personalized biological therapies, such as dual immune checkpoint inhibition (e.g., nivolumab plus relatlimab) [5] and chimeric antigen receptor (CAR) T-cell platforms [6,7]. While these approaches offer unprecedented therapeutic potential, they also introduce a substantial socioeconomic challenge for public health systems in middle-income countries.

The FNR has successfully guaranteed universal funding for generic TMZ, underpinning the 17-month survival baseline observed in this study [2]. As an oral, bioavailable medication, TMZ is a highly sustainable intervention. In contrast, advanced immunotherapies and autologous cellular platforms carry a substantial financial burden, often exceeding US$300,000-500,000 per patient globally.

Our findings show that the Uruguayan public health system can successfully deliver complex, standardized oncological protocols when universal funding is guaranteed. However, extending similar universal coverage to experimental biological therapies could threaten systemic financial stability; when advanced biotechnologies remain excluded from public formularies because of unfavorable cost-effectiveness ratios, health equity may be compromised. We therefore believe that a shift from fragmented, individual administrative decision-making toward regional, evidence-based health policy frameworks - supported by collaborative international pricing mechanisms and structured risk-sharing agreements - is warranted. Middle-income countries should use historical baselines, such as the one presented here, to inform participation in global clinical trial networks and to negotiate equitable access so that economic constraints do not determine patient survival in the immunotherapy era.

## Conclusions

This real-world evidence study shows that adherence to the frontline Stupp regimen confers a substantial survival benefit (24 vs. 11 months; p = 0.023) among patients with hemispheric GB treated within Uruguay’s public health system, with an overall cohort median survival of 17 months. These findings support the success of universal TMZ access policies. However, a persistent therapeutic ceiling remains, and the absence of historical molecular profiling highlights a critical regional diagnostic gap that should be addressed.

As the therapeutic landscape shifts toward high-cost immunotherapies and CAR-T platforms, regional health systems should use this historical baseline to inform agile, equity-driven funding policies and international pricing negotiations. Securing equitable access to advanced biotechnology will require moving beyond individual administrative allocation models toward structured, regional health policy frameworks.
